# 

**Published:** 1892-09-24

**Authors:** 


**Notices of Births, Deaths, and Marriages,** are inserted in this column at a charge of 2s. 6d. for an announcement not exceeding four lines.

### NOTICE TO CORRESPONDENTS.

All MS., Letters, Books for Review, and other matters intended for the Editor should be addressed The Editor, The Lodge, Porchester Square, London, W.

The Editor cannot undertake to return rejected M.S., even when accompanied by stamped directed envelope.

**Notice to Advertisers and Subscribers.**

All Advertisements, Orders for Copies of The Hospital, and Business Communications should be addressed to The Publisher, *not to the Editor*, The Hospital, 140, Strand, W.C.

The Cost of Subscription, post free, is as follows:—Per week, 2½d.; per quarter, 2s. 6d.; per half-year, 5s.; per annum, 8s. 6d. Foreign:—Per annum, 10s. 8d.; payable, in all cases, in advance.

**"Burdett's Hospital Annual," 1891—1892.**

Third year of publication. 600 pp. Boards, gilt lettering. A most complete guide to British and Colonial Hospitals, Dispensaries, Nursing and Convalescent Institutions, and Asylums. Price 3s. 6d. Published by The Scientific Press (Limited), 140, Strand, W.C.

**NOTICE.—The Index for Vol. XI. (ending March, 1892) is on sale at the Office of this Journal, price 2½d., post free.**

### SCRAPS AND GLEANINGS.

THE members of the Sanitary Congress last week visited Porchester Castle and Haslar Hospital.

A DISEASE peculiar to Japan is called the kake. It is believed to be the result of eating too much rice.

THE old baths at Montrose have been put into order so that if necessary they can be used as a cholera hospital.

A CASE of anthrax has occurred in a man admitted into the Royal Free Hospital. The sore was cut completely out, but the patient died of blood poisoning.

IT is stated that more than eighty-four years have elapsed since a ruling Queen of Prussia gave birth to a Princess, namely, Queen Louise, on February 1st, 1808.

DR. ADAMI, Fellow of Jesus College, Cambridge, has been appointed Professor of Pathology in the University of Montreal, Canada. The office has a salary of £800 a year.

ANOTHER fatal accident has occurred to an Alpine traveller, this time to a climber of German nationality. As in Mr. Nettleship's case the fatality was the result of a storm.

AT a meeting of the authorities of the Kingston Union and the Surbiton Commissioners it was decided to admit patients from Surbiton into the infectious hospital at Tolworth.

SEVERAL families living in the neighbourhood of Halebottom, Failsworth, have been attacked with small pox. Some of the sufferers have been removed to Monsall Hospital.

SCARLET FEVER has broken out in the Leyton district, and fifty-one cases have been reported to the Medical Officer. Thirty-nine of these have been traced to milk which came from one dealer.

A VERY insanitary state of affairs has revealed itself in Wednesbury, to the alarm of the inhabitants. It is found that large pools of highly offensive matter are in existence in close proximity to several cottages.

AT a meeting of the honorary members of the Sunday Concert Society, Mr. Edward Evans in the chair, it was decided to invite Lord Randolph Churchill, one of the honorary members, to accept the presidency of the society.

ACCORDING to the census returns for Ireland just issued, the total population in 1891 was 4,704,750 as against 5,174,836 in 1881. Great advance has been made in the housing of the people and good farm houses have increased by 10·5 per cent.

A NEW wing is being added to the St. Helen's Lantern Cottage Hospital including a special ward for women. The architect of the hospital, Mr. Thomas Beesley, of Warrington and Earlestown, has also supplied the plans for the new building.

A JOINT Infectious Diseases Hospital is to be erected near Dunfermline by the Dunfermline Local Authority and the Dunfermline District Committee of the Fife County Council at a cost of about £3,000. Messrs. Johnston and Burnie, Edinburgh, are the architects.

THE new cottage hospital for the use of non-infectious patients in Galashiels neighbourhood is to be erected in the Eastlands district. It is to contain fourteen wards, with other apartments and accessories for cooking and attendants. The total estimated cost is about £3,500.

THE governors of the West Bromwich District Hospital announced at their annual meeting that the new wards, which are sorely needed, will be ready for use in three months, but that the Board cannot open them until the annual subscriptions and donations are largely increased.

PART of the fund raised at the time of the Victoria Hall, Sunderland, disaster which occurred some years ago has been used to found the Heatherdene House Convalescent Home for Children, sent there from the Sunderland Infirmary. The Home is under the management of the Infirmary Directors.

THE new isolation wing of the Sunderland Infirmary, the gift of Miss Forster, of London and South Hetton, was opened by the Marchioness of Londonderry. The new building stands at the north-west corner of the Infirmary grounds, and is divided into two departments. The cost of the building is about £3,000.

THE lease of the house used as the convalescent home, Tradespark, Nairn, having fallen in, it has been found necessary to purchase a new site. A suitable one has been obtained not far from the former buildings, but subscriptions having fallen off, the committee are making an earnest appeal for funds to carry on the new building.

THE Manchester Board of Guardians have decided, at the request of Dr. Tatham, their Medical Officer, to offer additional facilities for vaccination. Dr. Tatham informed the Guardians that small-pox cases were rapidly occurring in widely separated parts of the city, and that a further spread of the disease was terrible to contemplate.

BEFORE the opening of the annual Sunday Hospital Concert, held at Barnsley, the Mayor, addressing the people, said that but for the valuable help given by the Hospital Saturday and Sunday Funds, the hospital would have been in debt to the amount of £815, and he expressed a wish that all would continue to support so valuable a cause.

THE Northern and Southern Hospitals, Liverpool, are in need of far greater support than they receive from the mercantile community. Close upon a thousand cases were relieved by the Ambulance last year, a fact which speaks for itself as to the relief given to dock and railway workers. The Northern Hospital is sorely in need of reconstruction, in spite of what has been done to supplement its deficiencies.

THE eleventh annual demonstration in aid of the Huddersfield Infirmary took place in the most favourable weather. The greatest attraction was the procession, and two of the most noteworthy exhibits were a miniature ship of wild flowers, the bottom of the waggon in which it was placed being covered with turf to represent the sea. The other was a canal boat, drawn by a rocking-horse, belonging to the Aird and Calder Navigation Company.

THE annual meeting of the directors of the Dunoon Cottage Hospital was held in the Burgh Buildings, when the report for the last year was read by the Secretary. Owing to want of funds the hospital had to be closed for eleven weeks, from April 25th to July 11th. The directors have not obtained sufficient funds to carry on the institution, and there is the sum of £4 7s. 6d. to their credit in the bank.

